# Untangling Community Assembly Through Functional Traits and Phylogenetic Alpha Diversity in Subtropical Karst Forests

**DOI:** 10.1002/ece3.71616

**Published:** 2025-06-26

**Authors:** Shichu Liang, Miao Dong, Yong Jiang, Daniel F. Petticord, Junwei Li, Jianghui Long, Quan Su

**Affiliations:** ^1^ Key Laboratory of Ecology of Rare and Endangered Species and Environmental Protection Guangxi Normal University Guilin China; ^2^ Cornell University Ithaca New York USA

**Keywords:** community assembly, competitive exclusion, environmental gradient, functional traits, karst forest types, phylogenetic alpha diversity

## Abstract

Taxonomic diversity is insufficient to fully characterize species co‐existence in forest ecosystems. Incorporating trait‐based and phylogenetic data into studies of communities provides insight into the mechanisms by which coexistence emerges and is maintained in forest landscapes. We integrated trait‐based and phylogenetic models of community structure to gain insight into community processes in deciduous, mixed, and evergreen forests. We also explored how phylogenetic community structure may vary across environmental gradients within deciduous, mixed, and evergreen forests using a null model approach. Plants appeared to transition from being acquisitive within dry, deciduous forest plots atop fertile soils to becoming more conservative in moist, infertile soil conditions in evergreen forests. We present this as strong evidence of environmental filtering mediating plant community composition. Our models suggest a strong influence of environmental filtering in deciduous forests and competitive exclusion and random processes in mixed and evergreen settings. Besides, thicker leaves, lower SLA, and higher WD in evergreen forests than deciduous forests may reflect adaptation to chronic herbivore pressure—a key biotic filter shaping trait distribution in different forest systems. Our results suggest that phylogeny and functional traits represent different lenses through which one may view community assembly, which should be viewed independently of each other. We suggest future research incorporating both phylogenetic and trait‐based perspectives, and possibly even more axes (stress tolerance, etc.) is necessary to provide insight into community assembly.

## Graphical abstract

Our study examines how functional traits and phylogenetic structure shape species coexistence in karst forests—ecosystems characterized by extreme soil heterogeneity, water scarcity, and nutrient limitations.

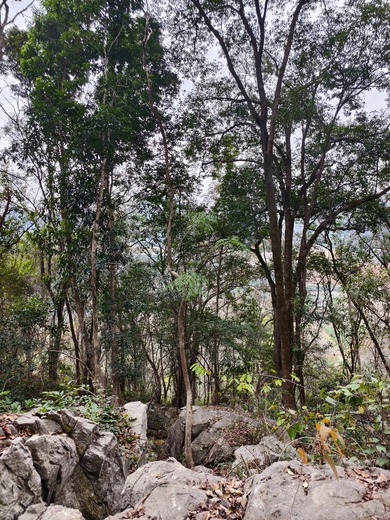

## 1 Introduction

If we assume that life is a constant struggle where natural selection constantly rarifies populations down through “selection of the fittest,” it can be difficult to understand how multiple species may coexist. Such a concept becomes even more challenging when we scale to the incredible diversity of forests; A 0.52‐km^2^ plot in Borneo may support up to 1175 tree species (LaFrankie 1995). Deterministic models of community assembly suggest that diversity is maintained because each individual species occupies a particular “niche” in which they may outcompete immediate competitors along some axis to ensure success (Tilman 1982; Tokeshi 1990; Wang et al. 2024). In such an approach, basic differences in the functional traits of species (rooting depth, fruiting time, leaf angle, etc.) produce fitness differences that allow for the fluctuating success of different heterospecifics over one another, maintaining diversity. In contrast, neutral theories suggest that diversity is the product of random change, namely ecological drift, historical inertia, or probabilistic dispersal. (Hubbell 2005). Understanding the balance and relative importance of these assembly processes remains a key unresolved question for many ecological communities (Alfonsi et al. 2023; Meng et al. 2024).

Generally, studies characterizing changes in vegetation along environmental gradients do so based on species identity (Bernard‐Verdier et al. 2012; Dong et al. 2020; Dani et al. 2023). Alternative approaches, such as trait‐based approaches, provide deeper insight into the deterministic ecological processes governing community assembly compared to analyses based solely on taxonomic classifications (Zakharova et al. 2019; Zhao et al. 2022; Funk et al. 2023; Liu et al. 2023).

For example, functional traits allow for interpretive work in understanding how species allocate resources for growth and reproduction—concepts such as the “fast‐slow” trait continuum rely on measures of certain functional traits such as specific leaf area (SLA), root length, or many other phenotypic measures (Reich 2014; Ma et al. 2024). A trait‐based approach to community assembly links species function to environmental conditions, providing mechanistic insight into coexistence, competition, and ecosystem processes beyond taxonomic identity (Jiang et al. 2024). For example, since traits and phylogeny are often linked through evolutionary history (Bello et al. 2017; Mertens et al. 2021) “phylogenetic conservatism” of trait(s) in a clumped group of closely related species, as observed in many forests, can be an indicator that a community was rarefied through habitat filtering, which enacted a strong selective pressure on the conservation of a trait or phenotypic motif. (Pyron et al. 2015; Nosrati et al. 2023). This information can be used to infer certain environmental pressures on how species have evolved. For example, in arid environments, plants may develop deep root systems or modified leaf structures to reduce water loss and improve water‐use efficiency, leading to a phylogenetically clustered community of functionally similar species with reduced phylogenetic alpha diversity (Rowland et al. 2023; Yang et al. 2023). Similarly, Han et al. (2023) found that in moist, stable evergreen forests, competition drives plants to optimize water and nutrient use by increasing leaf thickness (LT) and reducing SLA. This selective pressure results in a phylogenetically clustered distribution, where coexisting species exhibit similar trait adaptations despite their evolutionary relatedness.

Integrated approaches like these are invaluable for guiding conservation and restoration efforts. Identifying phylogenetic niche conservatism in degraded or threatened landscapes can help predict which plant species are likely to persist, informing targeted conservation strategies.

This is particularly relevant for ecosystems like karst landscapes, which support high levels of endemism yet face significant environmental pressures. While previous studies on karst ecosystems have examined vegetation restoration, soil characteristics, and taxonomic plant composition (He, Zhao, et al. 2021; Xue et al. 2022; Jian et al. 2025), relatively few have integrated functional traits with phylogenetic diversity. Given that karst flora plays a critical role in global biodiversity, understanding how evolutionary history and functional adaptations shape these plant communities is essential for effective conservation. Southern China and Southeast Asia host the world's largest humid tropical and subtropical karst landscapes, spanning over 800,000 km^2^ (Day and Urich 2000). These regions have long served as “natural laboratories” for ecological and evolutionary research, but are increasingly threatened by climate change and human activity (Clements et al. 2006). Recognizing their vulnerability, the International Union for the Conservation of Nature has identified karst landscapes worldwide as priorities for protection (Watson 1997).

Karst terrain forms through the dissolution of limestone and dolomite, resulting in a unique and distinctive terrain characterized by steep, irregular surfaces with rocky outcrops. Environmental variation driven by terrain heterogeneity—specifically, bare rock ratio (BRR), slope steepness, and soil thickness—reduces soil water retention capacity and destabilizes surface soil aggregates. The plant species inhabiting these ecosystems often exhibit mosaic distributions and have evolved specialized adaptations to survive under extreme conditions. These adaptations include well‐developed palisade tissue, small and thick leaves, and robust stems, all of which enhance resource acquisition in a nutrient‐ and water‐limited environment (Liu et al. 2020; Zeng et al. 2021).

Given these constraints, environmental filtering likely plays a dominant role in structuring karst plant communities by selecting for traits that enhance survival under harsh conditions. This process may drive the coexistence of closely related species with convergent trait strategies. To investigate this, we examined 3 natural forest types—deciduous, mixed, and evergreen forests—using data from 4 functional traits and 12 environmental variables to assess community structure. Specifically, we addressed the following questions: (1) How do plant functional traits and adaptation strategies vary across different forest types? (2) How does phylogenetic community structure respond to environmental gradients within these forests? By addressing these questions, this study aims to provide new insights into transitional assembly processes in subtropical karst forests, contributing to broader community assembly theory. Additionally, our findings will offer a scientific basis for guiding vegetation restoration and ecological reconstruction efforts in the karst hills region.

## 2 Materials and Methods

### 2.1 Study Area

This study was conducted in the Guilin karst hills region, located in the northeast of Guangxi Zhuang Autonomous Region, China (110°14′–110°42′ E, 24°43′–25°20′ N). The elevation ranges from 180 to 240 m, and the area is classified as a typical karst rock landscape (He, Yan, et al. 2021). It experiences a subtropical monsoon climate, with an average annual temperature of 17.8°C–19.1°C and a frost‐free period of 309 days (China Meteorological Data Service Center; http://data.cma.cn). The coldest month (January) averages 7.9°C, and the mean annual precipitation is 1856.7 mm. The topography consists of limestone valleys flanked by steep hills, with rugged terrain and slopes ranging from 15.06° to 59.38° over 20 m grid cells. The underlying rock is primarily limestone interbedded with dolomite, which is rich in calcium. Soil, where present, is thin and generally tends to accumulate only in rock fractures.

We identified three distinct vegetation types within the karst landscape, each shaped by different environmental gradients (Table 1, Table S1, Figures S1 and S2): (1) *Deciduous Broad‐Leaved Forests (Deciduous Forests)*—These forests occur in areas with higher soil nutrient content, lower water availability, and a relatively high proportion of exposed rock. They also have the thickest soil layer among the three forest types. Dominant tree species include *Celtis sinensis, Mallotus repandus, Choerospondias axillaris, Chimonanthus nitens, Cornus wilsoniana*, and *Boniodendron minus* (Table S1). (2) *Mixed Evergreen and Deciduous Broad‐Leaved Forests (Mixed Forests)*—Found in habitats with moderate soil nutrient content, the highest rock exposure, and relatively shallow soil. These forests support a mix of evergreen and deciduous species, including *Quercus glauca, Zelkova schneideriana, Boniodendron minus, Mallotus philippensis, Cinnamomum saxatile*, and *Pittosporum planilobum* (Table S1). (3) *Evergreen Broad‐Leaved Forests (Evergreen Forests)*—These forests are distributed in areas with the poorest soil conditions, lowest nutrient availability, and a lower proportion of exposed rock. They typically occur on flatter slopes and have the thickest soil layer of the three types. Dominant species include *Quercus glauca, Mallotus philippensis, Pittosporum planilobum, Decaspermum parviflorum, Albizia julibrissin*, and *Murraya exotica* (Table S1).

**TABLE 1:** Basic plot information in three forest types.

Parameter	Deciduous forests	Mixed forests	Evergreen forests
Number of individual deciduous/evergreen trees	1752/1188	862/779	2660/4341
Number of deciduous/evergreen species	36/27	42/49	44/40
Soil thickness (cm)	28.88 ± 10.08	19.40 ± 10.46	31.66 ± 13.64
Elevation (m)	184–228	179–244	195–240
Slope (°)	15.06–59.38	24.71–47.63	17.96–45.88

### 2.2 Field Sampling

Field surveys were conducted in three separate periods: July–September in 2019, 2021, and 2022. A total of 25 plots (20 × 20 m each) were randomly selected across different sections within the same slope conditions, ensuring a minimum buffer distance of 10 m between adjacent plots for each forest type. The study sites were located in Mao Village (Lingchuan County), Long Village (Yongfu County), and Beitou Village (Yangshuo County). Each 20 × 20 m plot was subdivided into four 10 × 10 m subplots for vegetation and environmental surveys. Vegetation surveys and soil sampling were conducted from July to August. To assess species abundance and diversity, all free‐standing woody trees with a diameter at breast height (DBH) ≥ 1 cm at 1.3 m were measured, tagged, and identified in each plot. For trait measurements, leaf samples were collected from each tree. Leaves were not necessarily sun‐exposed, as many species achieve maximum leaf size in the understory without full sunlight exposure. However, for trees with a canopy height ≥ 10 m, sun‐exposed branches were sampled using a high‐branch pruning tool. For understory species, leaves were selectively collected from the uppermost sections of the plants (Perez‐Harguindeguy et al. 2016). Each tree was sampled for three leaves and three stems. Collected samples were sealed in plastic bags, placed into a large woven polypropylene bag, and transported to the laboratory within 12 h for further analysis.

### 2.3 Trait Data Measurement

We measured 4 functional traits for all woody individuals, representing 150 species across the 3 forest types, following standard protocols (Perez‐Harguindeguy et al. 2016). These traits included DBH (cm), LT (mm), SLA (cm^2^/g), and wood density (WD, g/cm^3^). These traits were chosen because they represent key axes of plant functional strategy quantitatively (Wright et al. 2004). DBH was measured at 1.3 m using a flexible measuring tape, recorded to the nearest 0.1 cm, and converted into diameter using the standard circumference‐to‐diameter formula (de Souza et al. 2013). Trees with larger DBH typically have wider crowns and greater foliage, and often possess deeper root systems (Guo et al. 2013). SLA was calculated as leaf area per unit of dry leaf mass. Leaf area was determined using a YMJ‐C scanner (Tuopu, Zhejiang, China) with an integrated measurement system. The leaf samples were dried at 65°C for 48 h until a constant weight was reached. The final dry mass was then recorded with a precision of 0.1 mg. SLA is a key indicator of a plant's ability to utilize resources efficiently and adapt to environmental stress (Garnier et al. 2001; Wright et al. 2004). LT was measured using a caliper with a precision of 0.05 mm, with three replicates per sample. LT is associated with leaf longevity and water storage capacity, both of which are critical traits for plants growing in resource‐limited environments (Perez‐Harguindeguy et al. 2016). WD was assessed for branches approximately 1 cm thick and 10 cm long. Bark and pith were removed before measuring volume using the water displacement method. Samples were then oven‐dried at 80°C to obtain dry weight. WD was calculated as dry weight divided by volume (Cornwell et al. 2006; Ogasa et al. 2013). WD is a key trait influencing xylem water transport, drought tolerance strategies, and mechanical support (De Guzman et al. 2020; Garcia et al. 2021; Carvalho et al. 2023).

### 2.4 Measurement of Environmental Factors

Soil samples were collected from the four corners and the center of each plot, resulting in five subsamples per plot. Before sampling, surface debris, including plant material and roots, was removed. Soil was then extracted from a depth of 0–20 cm. Fresh soil samples were immediately placed into pre‐labeled plastic bags and transported to the laboratory. Each 1000 g soil sample was air‐dried and sieved to 1 mm for physical analyses and 0.15 mm for chemical analyses. Soil properties were measured following standard agricultural and chemical protocols (Xia et al. 2023). The variables analyzed included soil water content (SWC, %), soil pH, total nitrogen (TN, g/kg), available nitrogen (AN, mg/kg), available phosphorus (AP, mg/kg), water‐soluble calcium (Ca, g/kg), and total phosphorus (TP, g/kg). To improve accuracy, three replicate measurements were taken for each soil parameter, and the mean value was used in subsequent analyses.

In addition to soil properties, we recorded various environmental factors for each plot. Elevation and slope were automatically acquired using the JRBP Geographic Information System (GIS). Soil thickness was measured using a 1.5 m steel probe, which was hammered into the parent rock at each soil sampling location. Understory irradiance was estimated using hemispherical canopy photographs taken at 1.5 m above ground level in each 10 × 10 m subplot. Photographs were captured using a fisheye lens mounted on a Nikon D80 camera and analyzed using the Gap Light Analyzer software (Frazer 1999) to determine canopy cover. Canopy openness (CO, %) was calculated as 1 − the proportion of closed‐canopy pixels. In deciduous forests, CO was estimated during the period of leaf fall, while for mixed and evergreen forests, it was measured during the growing season. BRR (%) was visually estimated by assessing the proportion of exposed rock relative to the total ground surface within each 10 × 10 m subplot. This metric was used to evaluate the extent of rock outcrops, which influence soil depth and water availability in karst landscapes.

### 2.5 Statistical Analyses

#### 2.5.1 Shifts in Community‐Level Mean Trait Across Different Forest Types

To assess the functional responses shaping community assembly in different forest types, we calculated the community‐weighted mean (CWM) for each functional trait. This approach incorporated two datasets: a species‐trait matrix and a plot‐species abundance matrix. These two matrices were integrated to generate a plot‐trait matrix, enabling the calculation of CWM values for forest communities. The CWM metric, which accounts for species abundance, provides a more ecologically meaningful representation of trait variations compared to the unweighted mean. By incorporating species abundance, CWM better captures trait filtering processes and highlights the ecological mechanisms structuring plant communities. The CWM for each trait was computed using the following equation:

$$\mathrm{CWM}=\sum_{i=1}^{S}W_{i}\times X_{i}$$

where *S* represents species number at a plot within each forest type, *W*_*i*_ is the relative abundance of species *i*, and *X*_*i*_ is the functional trait value of species *i* at each plot within each one (Jiang et al. 2015). We utilized the FD function in the “dbFD” package (Grenié and Gruson 2023) to compute the community‐level functional trait values. Subsequently, we assessed differences in community‐level mean trait values and soil environment factors between two groups of sampling units using one‐way ANOVA with least significant difference method (LSD) test at a significance level of *p* < 0.05.

#### 2.5.2 Phylogenetic Tree Construction

We compiled a species list encompassing all 150 species collected from 104 genera and 47 families. Botanical nomenclature was standardized following The Plant List (TPL, version 1.1, www.theplantlist.org). Using this standardized dataset, a synthetic phylogenetic tree was constructed with the mega‐tree function (scenario 3) in the R package “V.PhyloMaker” (Jin and Qian 2019). This method allows for the integration of species into a phylogenetic framework while accounting for unresolved relationships in the tree topology.

#### 2.5.3 Phylogenetic Signal

To evaluate whether the functional traits exhibit a phylogenetic signal, we calculated Blomberg's *K* statistic for each of the four traits across the three forest types (Blomberg et al. 2003). A *K* value of 0 indicates the absence of phylogenetic signal, meaning traits are randomly distributed across the phylogeny. A *K* < 1 suggests that closely related species are less similar than expected, while a *K* > 1 implies that closely related species exhibit greater trait similarity than predicted under Brownian motion evolution. The statistical significance of the phylogenetic signal was tested by comparing observed *K* values against a null distribution generated by randomly permuting species names (999 iterations) along the phylogenetic tree (*p* < 0.05). The phylogenetic signal was computed for all species and separately for angiosperms within each forest type. Analyses were conducted using the multiPhylosignal function from the “picante” R package (Kembel et al. 2010; E‐Vojtkó et al. 2023).

#### 2.5.4 Phylogenetic Alpha Diversity

To quantify phylogenetic relatedness among woody species in each plot, we calculated the Net Relatedness Index (NRI) and Nearest Taxon Index (NTI) (Webb 2000; Kraft et al. 2007). NRI measures the standardized effect size of the mean phylogenetic distance (MPD), which estimates the average phylogenetic distance among all taxa in a community. NTI assesses the phylogenetic distance to the most closely related co‐occurring taxon, based on the mean nearest taxon distance (MNTD). To generate null expectations, randomized communities (*n* = 999) were simulated by randomly drawing species from the regional species pool while maintaining observed species richness and occurrence frequencies. The species pool included all 150 species recorded in the study region. NRI and NTI were calculated using the following equations:

$$\mathrm{NRI}=-1\frac{{\mathrm{MPD}}_{\text{observed}}-{\mathrm{MPD}}_{\text{randomized}}}{{\text{sdMPD}}_{\text{randomized}}}$$

$$\mathrm{NTI}=-1\frac{{\text{MNTD}}_{\text{observed}}-{\text{MNTD}}_{\text{randomized}}}{{\text{sdMNTD}}_{\text{randomized}}}$$

where MNTD_observed_/MPD_observed_ are the observed MNTD/MPD, MNTD_randomized_/MPD_randomized_ is the expected MNTD/MPD of the randomized communities (*n* = 999) and sdMNTD/MPD_randomized_ is the standard deviation of the MNTD/MPD for the randomized communities.

Positive values of NRI and NTI indicate phylogenetic clustering, meaning that species in a community are more closely related than expected under random assembly. Negative values indicate phylogenetic evenness (overdispersion), suggesting that species are more distantly related than expected. Phylogenetic metrics were computed using the “picante” R package (Kembel et al. 2010; E‐Vojtkó et al. 2023). To determine whether the phylogenetic structure (NRI/NTI) of the plots deviated significantly from the expected null value of 0, we applied a two‐tailed Wilcoxon signed‐rank test for each forest type.

## 3 Results

### 3.1 Species Richness

**FIGURE 1:**
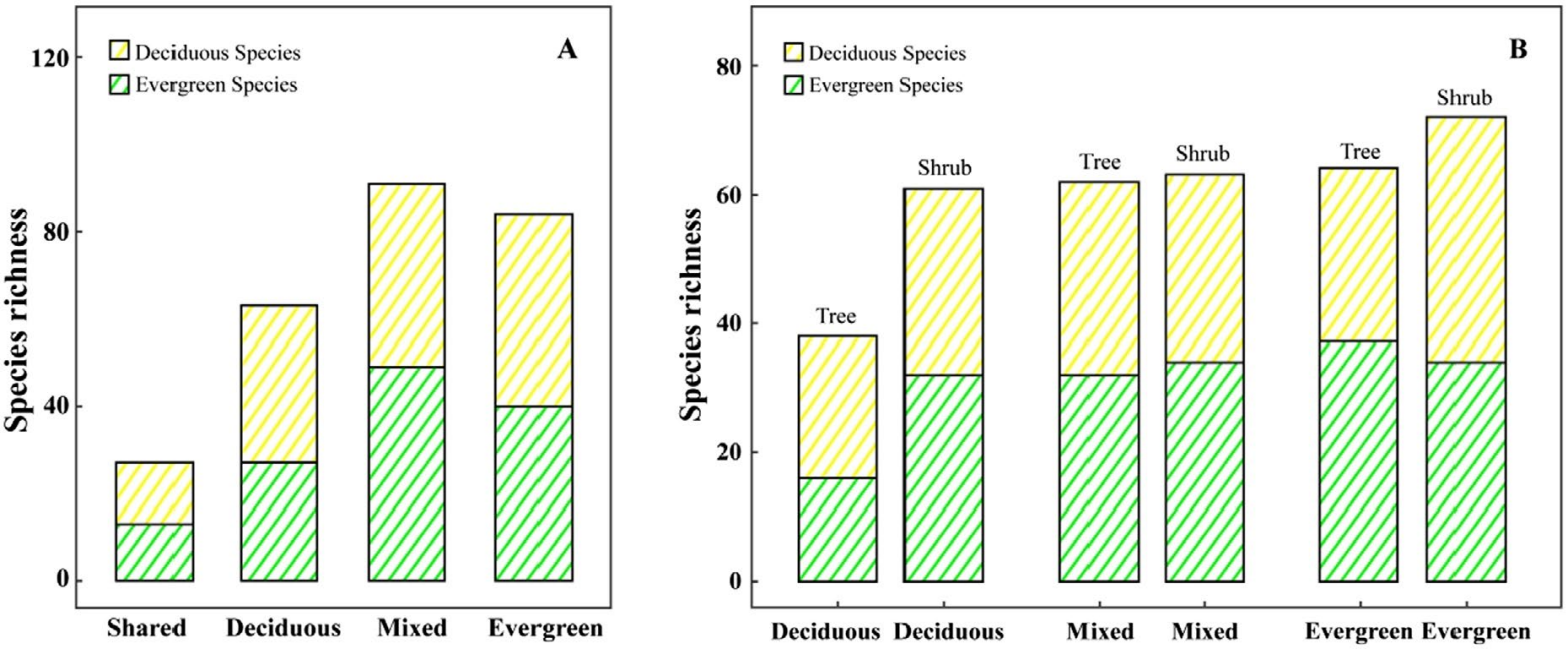
Basic species information at different forest types. (A) Species richness of the three forest types. (B) Composition of evergreen and deciduous species in the three forest types.

In total, we sampled 11,546 woody stems representing 150 species, 104 genera, and 47 families. Based on species relative density, basal area, and frequency, 27 species were found in all three forest types, while 63, 90, and 84 species were restricted to the deciduous, mixed, and evergreen forests, respectively (see Table S1, Figure 1). Analysis using the Jaccard similarity index suggests each forest type is distinct (Table 2).

**TABLE 2:** The Jaccard similarity index for the three forests.

	Deciduous forests	Mixed forests
Mixed forests	0.38	
Evergreen forests	0.54	0.49

Deciduous, mixed, and evergreen forests contained 36, 42, and 44 evergreen species and 27, 49, and 40 deciduous species, respectively. In the shrub layer, we identified 32, 34, and 34 evergreen species, along with 29, 29, and 38 deciduous species in the deciduous, mixed, and evergreen forests, respectively.

### 3.2 Changes on Soil Environmental Factors

**FIGURE 2:**
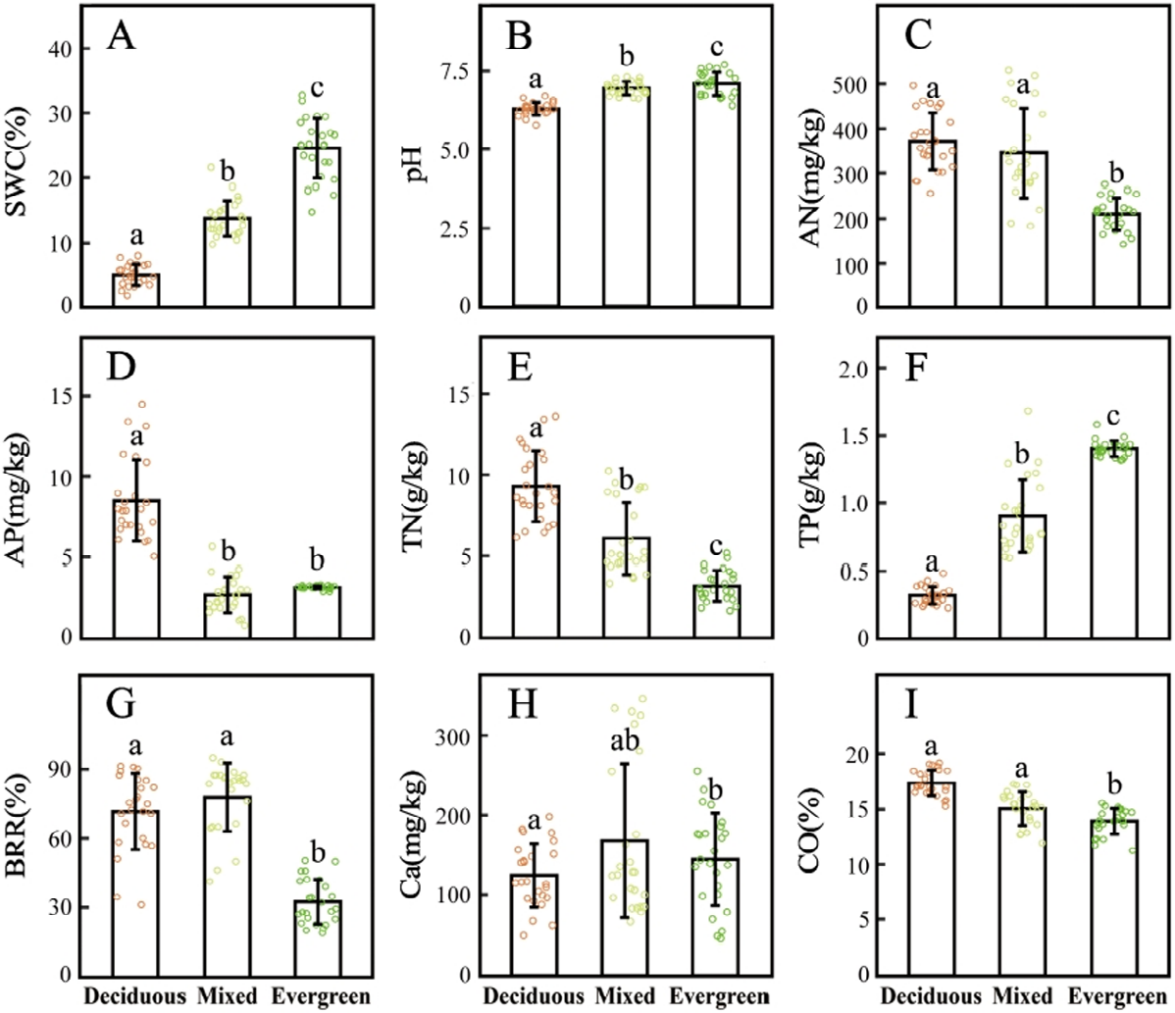
Differences in (A) SWC, (B) pH, (C) AN, (D) AP, (E) TN, (F) TP, (G) BBR, (H) Ca, and (I) CO among the three forest types. AN, available nitrogen; AP, available phosphorus; BBR, bare rock ratio; Ca, water‐soluble calcium; CO, canopy openness; pH, soil pH; SWC, soil water content; TN, total nitrogen; TP, total phosphorus. Bars indicate (mean ± SD). The distinct letters of a‐c stand for significant difference among three forest types (*p* < 0.05).

Several soil factors differed significantly among the deciduous, mixed, and evergreen forests (Figure 2). Specifically, SWC, pH, TN, and TP varied significantly across the three forest types (*p* < 0.05; Figure 2A,B,E,F). AN, BRR, and CO were significantly lower in evergreen forests compared to deciduous and mixed forests (Figure 2C,G,I). Calcium (Ca) concentrations in deciduous and evergreen plots were lower than in mixed forests, with a significant difference observed between deciduous and mixed forests (Figure 2H).

AP was significantly higher in deciduous forests compared to mixed and evergreen forests, though no significant difference was found between the latter two (Figure 2D).

### 3.3 Changes in Community‐Weighted Mean Traits

**FIGURE 3:**
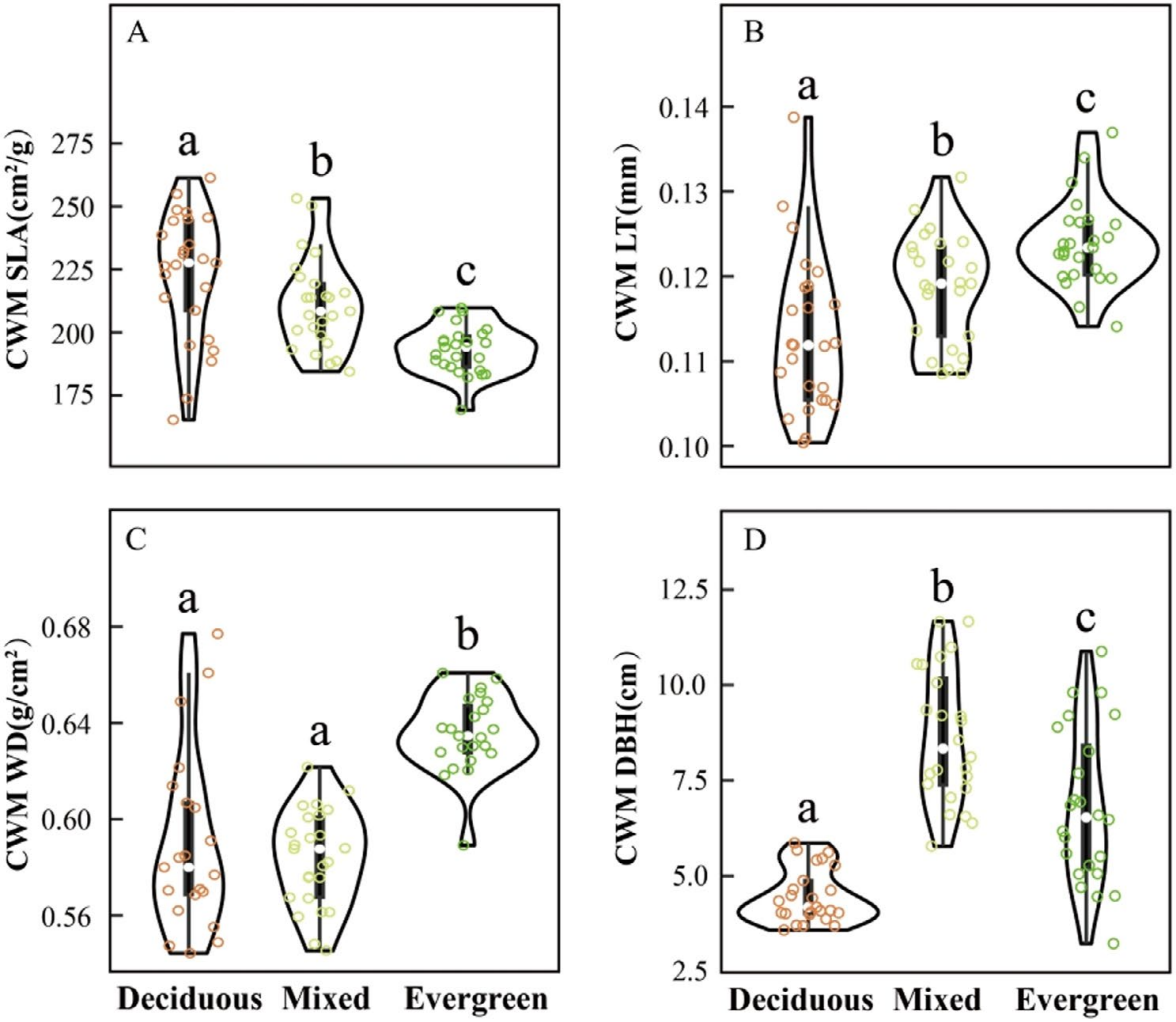
Community‐weighted means values of four plant functional traits (A) CWM SLA, (B) CWM LT (C) CWM WD and (D) CWM DBH in three forest types. DBH: diameter at breast height; LT: leaf thickness; SLA: specific leaf area; WD: wood density. The distinct letters of a–c stand for significant difference among three forest types (*p* < 0.05).

Overall, we observed shifts in CWM trait values across the forest gradient from deciduous to evergreen forests (Figure 3). CWM SLA significantly decreased, while CWM LT significantly increased, reflecting trait responses to varying environmental gradients among the three forest types.

CWM DBH was lowest in deciduous forests and highest in mixed forests. No significant difference was observed in CWM WD between deciduous and mixed forests, though both differed significantly from evergreen forests (Figure 3C).

### 3.4 Phylogenetic Signal of Functional Traits

All assessed traits had Blomberg's *K* values > 0 but < 1, indicating weak but detectable phylogenetic signals. Among the four traits across the three forest types, some exhibited statistically significant *K* values (*p* < 0.05), with values ranging from 0.180 to 0.475 (Table 3).

**TABLE 3:** The phylogenetic signal intensity of functional traits in three forest types.

Functional traits of tree species	Deciduous forests		Mixed forests		Evergreen forests	
	Blomberg's *K*	*p*	Blomberg's *K*	*p*	Blomberg's *K*	*p*
Wood density (WD; mg/cm^3^)	0.475	0.029*	0.322	0.008**	0.350	0.019*
Leaf thickness (LT; cm)	0.461	0.034*	0.309	0.018*	0.290	0.181
Specific leaf area (SLA; cm^2^/g)	0.475	0.025*	0.260	0.124	0.210	0.686
Diameter at breast height (DBH; cm)	0.411	0.003*	0.132	0.706	0.180	0.752

* *p* < 0.05.

** *p* < 0.01.

In deciduous forests, WD, LT, SLA, and DBH had *K* values significantly > 0 but < 1, suggesting a weak yet significant phylogenetic signal. However, in mixed and evergreen forests, LT, SLA, and DBH showed no significant phylogenetic signal, whereas WD exhibited a strong phylogenetic signal, indicating that WD was more conserved among closely related species in these two forest types.

### 3.5 Phylogenetic Alpha‐Diversity

**FIGURE 4:**
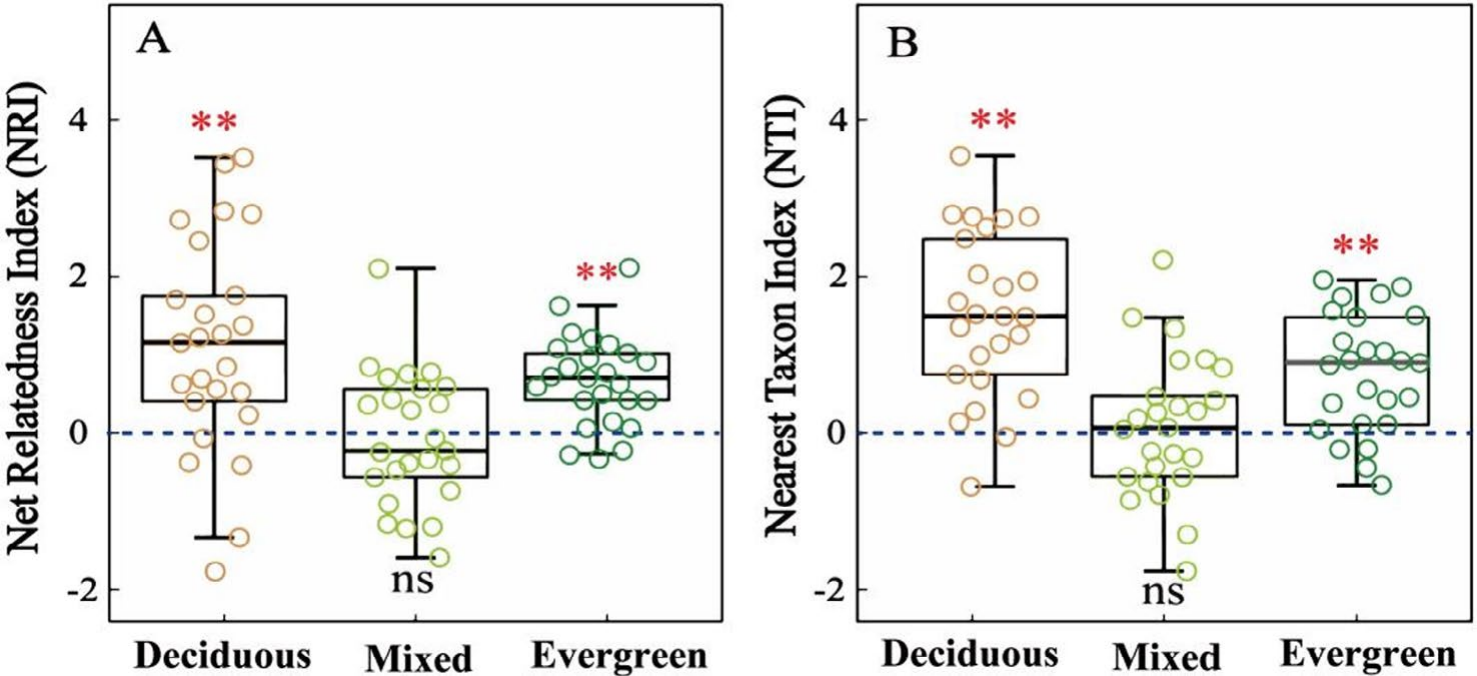
Phylogenetic alpha diversity in three forest types. Comparison of (a) Net Relatedness Index (NRI) and (b) Nearest Taxon Index (NTI) in three forest types. Positive values indicate clustering, whereas negative values indicate over‐dispersion, and 0 indicates random. Asterisks indicate overall significance according to the two‐tailed Wilcoxon signed‐ranks test (**p < 0.01; ns: no significant difference).

Figure 4 showed the NRI and NTI were significantly > 0 in deciduous and evergreen forests, indicating significant phylogenetic clustering in these communities. In contrast, NRI and NTI were approximately 0 in mixed forests, suggesting a phylogenetically random species distribution in this forest type.

## 4 Discussion

### 4.1 CWM_traits_ Response to Environmental Conditions at Different Forest Types

In this study, we observed that most community‐level functional traits shift in response to SWC, soil nutrient availability, topography (such as BRR, slope, and soil thickness), and light conditions across the three forest types (Figures 2 and 3, Figures S3 and S5). Previous studies have demonstrated that SLA serves as a reliable proxy for understanding how different tree species adopt varying ecological strategies for resource acquisition and utilization (Chu and Farrell 2022; Holden and Cahill 2024; Pantaleão et al. 2024). Our findings show that deciduous forests exhibit the highest CWM SLA, whereas evergreen forests display the lowest values. SLA is often associated with high resource acquisition; plants invest in bigger leaves to get more sunlight, which implies other resources are in sufficient abundance to (a) make more leaf tissue, and (b) to push plants toward competing for C as opposed to N or P. This concept is in line with the general idea that deciduous species tend to thrive in resource‐rich environments by adopting an acquisitive strategy, whereas evergreen species persist in resource‐limited environments through a conservative strategy (Lavorel and Garnier 2002; Garnier et al. 2007; Firn et al. 2019). So the lower SLA values of evergreen forests may reflect a coordinated defense strategy—high dry leaf mass implies higher structural investment which may enhance drought tolerance, increase the potential that nutrients are conserved and recycled during decomposition, and reduce palatability to herbivores (Dirzo and Boege 2008).

CWM LT showed an increasing trend from deciduous to mixed to evergreen forests, indicating that plants adjust leaf traits to store water and nutrients in response to topographic constraints such as BRR and slope (Li et al. 2022; Wang et al. 2022) (see Figure S3). The lowest LT values were observed in deciduous forests, where species exhibit drought‐adaptive strategies to enhance water‐use efficiency by reducing LT, particularly in areas with low SWC. In contrast, evergreen species, which displayed the highest LT, exhibit greater leaf hydraulic conductance and water storage capacity, helping to prevent resource depletion and extend leaf lifespans (Huang et al. 2018). Also, the observed LT patterns suggest an evolutionary preference for constitutive structural defenses against persistent herbivory pressure. Evergreen forests, characterized by stable canopy conditions and year‐round foliage, experience continuous herbivore activity. This persistent pressure likely favors increased LT as a folivore deterrent strategy (Dirzo and Boege 2008). Moreover, thick‐leaved species (e.g., evergreen plants) often couple structural defenses with chemical defenses, allocating additional resources to secondary metabolites (e.g., phenolics, tannins). These compounds synergistically enhance resistance by reducing leaf palatability, disrupting herbivore digestion, or exerting toxic effects (Agrawal and Fishbein 2006). The distribution pattern of CWM WD was lowest in deciduous forests, where light availability is high, soil nutrients are abundant, and SWC is relatively low. Species in these habitats have evolved a fast‐growth, short‐lifespan strategy, prioritizing rapid resource acquisition over structural investment (Nawaz et al. 2023; Xu et al. 2023). Conversely, the highest CWM WD values were observed in evergreen forests, where soil nutrients (AP, AN, and TN) and CO were significantly lower than in the other two forest types. High WD is often linked to survival strategies in nutrient‐poor environments, as it provides mechanical support, greater resistance to environmental stress, and improved water transport efficiency (Maynard et al. 2022; Castillo‐Figueroa et al. 2023). Additionally, dense wood serves as an effective defense against wood‐boring insects (e.g., moths) and large herbivores while contributing to the extended lifespans characteristic of evergreen species. It is also possible that increasing WD reflects the higher proportion of carbon available for storage in ecosystems where C is more available than either N or P, as we would anticipate in these nutrient‐poor settings. CWM DBH was lowest in deciduous forests, likely due to the predominance of small trees and shrubs, as well as ecological constraints that limit DBH expansion (see Table S2; Subedi et al. 2019). A slightly higher CWM DBH was observed in evergreen forests, where lower availability of soil nutrients, reduced light availability, and high tree density restrict the growth potential of some individuals, resulting in smaller diameter classes (Figure 3D; see Table S2). In contrast, CWM DBH was highest in mixed forests, likely due to niche differentiation among coexisting tree species, which allows for more efficient resource partitioning (Li et al. 2019; Zhang et al. 2023).

These findings highlight a gradual shift in community‐level functional traits along environmental gradients in the three forest types. Specifically, they illustrate a transition from fast‐growing, resource‐acquisitive strategies in deciduous forests to resource‐conservative, stress‐tolerant strategies in evergreen forests. Moreover, our results underscore the strong role of environmental filtering in shaping community assembly, particularly as forest composition shifts from deciduous to evergreen species in response to increasing resource limitations.

### 4.2 Phylogenetic Alpha Diversity Changes Across Three Forest Types

The significant positive values of NRI and NTI in deciduous and evergreen forests—with some plots exceeding 1.96—suggest that woody plants in these communities are phylogenetically clustered (Figure 4). Also, we found greater variation in NRI and NTI when comparing across deciduous forests than evergreen forests. This pattern may be driven by higher micro‐environmental heterogeneity in deciduous forests, where complex topography (e.g., steep slopes and high CO and BRR) creates pronounced gradients in soil water and nutrient availability (Figure 2). This environmental heterogeneity appears to drive spatially variable environmental filtering across plots, with different microhabitats selecting for phylogenetic clusters of species adapted to local conditions (e.g., drought‐tolerant lineages on south‐facing slopes). Such habitat‐specific filtering likely accounts for the broader range of phylogenetic clustering patterns observed across deciduous forest plots relative to the more homogeneous evergreen communities.

However, the inconsistent phylogenetic signal observed across the three forest types indicates that the measured traits are not entirely phylogenetically conserved and tend to exhibit trait convergence to some extent. The variation in trait evolution under different environmental conditions suggests that community assembly mechanisms differ considerably across forest types. In deciduous forests, the traits WD, LT, SLA, and DBH exhibited significant, yet weak phylogenetic signals (*K* > 0 but < 1), implying that these traits are influenced by evolutionary history within this habitat.

When combined with the observed phylogenetic clustering (Figure 4A), this suggests that environmental filtering may be the primary force structuring the community. Environmental filtering tends to promote the coexistence of closely related species, which share similar functional traits or survival strategies. For instance, deciduous species in this habitat may reduce LT to minimize water loss or increase SLA to enhance light capture and soil nutrient acquisition, allowing them to adapt to seasonal droughts and cold stress (Lima et al. 2021; Medeiros et al. 2024). Additionally, Swenson and Enquist (2009) found that small‐diameter plants in a Neotropical dry forest tend to be more phylogenetically clustered, a pattern also observed in the deciduous forests of this study. Many small‐diameter individuals belonged to closely related species (see Table S2), likely due to niche similarity and limited resource availability. This clustering may be further influenced by local topographic factors, such as high BRR and steep slopes, which restrict water availability and intensify environmental filtering in deciduous forests.

In mixed forests, only WD and LT exhibited a significant phylogenetic signal, and generally the phylogenetic structure of the community was evenly distributed around the null expectation (Figure 4). This pattern suggests that both phylogenetic clustering and overdispersion may be occurring in this community, reflecting a transitional community structure. Notably, we observed a higher proportion of small‐diameter individuals compared to medium and large trees in this habitat (see Table S2). Small‐diameter species typically exhibit significant phylogenetic clustering (Li et al. 2022), but as trees grow, competition increases, leading to greater spatial separation among larger individuals, which in turn promotes phylogenetic divergence. Our findings align with studies suggesting that phylogenetic randomness arises from the dynamic interplay between habitat filtering and competitive exclusion.

These opposing forces eventually balance each other, shaping the evolutionary landscape in complex ways (Mayfield and Levine 2010; Kremer and Klausmeier 2013). Moreover, the mixed forests represents a transitional stage between deciduous and evergreen forests (Sánchez de Dios et al. 2023), where environmental conditions are continuously changing. This transition phase may accelerate the decline of shade‐intolerant deciduous species, leading to the gradual opening of vegetation gaps. Such gaps create opportunities for late‐colonizing evergreen species, which may arrive randomly through dispersal events, contributing to the high species turnover in mixed forests. This suggests that chance processes, such as random extinction, colonization, and dispersal, are likely to significantly influence the species composition of mixed forests.

In evergreen forests, both NRI and NTI indicated phylogenetic clustering, yet only WD exhibited a significant phylogenetic signal, while LT, SLA, and DBH did not. The lack of significant phylogenetic signals in LT, SLA, and DBH suggests that these traits may be more labile across phylogenetically diverse species, allowing for a wider range of trait values to be present within the community. This suggests that competitive exclusion (i.e., trait convergence), rather than environmental filtering, is the primary driver of community assembly in evergreen forests. It was evident among species from Euphorbiaceae, Fagaceae, and Leguminosae in evergreen forests, which displayed significant differences in SLA, LT, and DBH despite their distant evolutionary histories (see Figure S4). Compared to the other two forest types, evergreen forests are characterized by higher SWC, lower rock exposure, flatter slopes, and thicker soil layers. These conditions favor plant survival and growth, reducing the relative importance of environmental constraints. Furthermore, the year‐round herbivore pressure in evergreen forests may act as a chronic biotic stress, selecting for defensive traits with different phenotypic plasticity (i.e., thicker leaves, lower SLA and higher WD comparing to deciduous forests) that contribute to phylogenetic clustering by filtering species with divergent strategies (Dirzo and Boege 2008). This biotic context reinforces our interpretation that traits distribution patterns represent adaptive responses to biotic stressors. Taken together, our results suggest that deciduous forests are primarily shaped by environmental filtering, mixed forests exhibit a balance between habitat filtering and competitive exclusion, and evergreen forests are structured predominantly by competitive interactions, trait convergence and to some extent biotic (herbivory pressure) stressors. These findings provide valuable insights into the ecological and evolutionary processes driving community assembly in karst forest ecosystems.

## 5 Conclusions

In this study, we quantified CWM traits and phylogenetic alpha diversity in woody plant assemblages across three forest types. Our findings revealed that community‐level functional traits varied in parallel with changes in SWC, nutrient availability, topography, and light conditions across diverse environmental gradients. Notably, we observed a gradual transition from fast‐growing strategies to resource‐conservative adaptations, emphasizing the strong role of environmental filtering in shaping community assembly, particularly in the transition from deciduous to evergreen forests where resources become increasingly limited. This transition was further mediated by biotic interactions, especially herbivory pressure. Evergreen forests, with their year‐round leaf retention, face chronic herbivore pressure that selects for constitutive defenses such as thicker leaves, lower SLA and higher WD. These traits not only deter herbivores but also align with the extended lifespans and slower resource acquisition strategies typically prescribed to evergreen species.

Patterns of phylogenetic clustering and phylogenetic randomness varied across the three forest types. However, given the inconsistent phylogenetic signal among the measured traits, we conclude that phylogenetic clustering may arise from different assembly mechanisms, such as environmental filtering or competitive exclusion. These results highlight the utility of phylogenetic signal analysis in detecting trait evolution and understanding the processes underlying community assembly.

By applying the methods described in this study, we found that phylogenetic structure and functional traits are, to some extent, independent of each other. This suggests that relying on a single approach may not be sufficient to draw definitive conclusions about community assembly dynamics. Therefore, integrating both phylogenetic and trait‐based perspectives, along with additional approaches, is essential for developing a comprehensive understanding of the ecological and evolutionary processes shaping plant communities.

## Author Contributions

**Shichu Liang:** supervision (equal), writing – review and editing (equal). **Miao Dong:** conceptualization (equal), data curation (equal), formal analysis (equal), writing – original draft (equal). **Yong Jiang:** funding acquisition (equal), project administration (equal), writing – review and editing (equal). **Daniel F. Petticord:** writing – review and editing (equal). **Junwei Li:** supervision (equal), writing – review and editing (equal). **Jianghui Long:** data curation (equal), formal analysis (equal). **Quan Su:** supervision (equal), writing – review and editing (equal).

## Conflicts of Interest

The authors declare no conflicts of interest.

## Supporting information


Appendix S1.

Appendix S2.

Appendix S3.

Appendix S4.

## Data Availability

All the required data are uploaded as Supporting Information S1.

## References

[ece371616-bib-0001] Agrawal, A. , and M. Fishbein . 2006. “Plant Defense Syndromes.” Ecology 87, no. sp7: S132–S149. 16922309 10.1890/0012-9658(2006)87[132:pds]2.0.co;2

[ece371616-bib-0002] Alfonsi, E. , M. Benot , and D. Alard . 2023. “Processes Underlying the Assembly of Plant Communities: The Role of Environmental Filtering Heterogeneity in a Competition–Dispersal Experiment.” Journal of Vegetation Science 34, no. 2: e13186.

[ece371616-bib-0003] Bello, F. , P. Šmilauer , J. A. Diniz‐Filho , et al. 2017. “Decoupling Phylogenetic and Functional Diversity to Reveal Hidden Signals in Community Assembly.” Methods in Ecology and Evolution 8, no. 10: 1200–1211.

[ece371616-bib-0004] Bernard‐Verdier, M. , M. Navas , M. Vellend , C. Violle , A. Fayolle , and E. Garnier . 2012. “Community Assembly Along a Soil Depth Gradient: Contrasting Patterns of Plant Trait Convergence and Divergence in a Mediterranean Rangeland.” Journal of Ecology 100, no. 6: 1422–1433.

[ece371616-bib-0005] Blomberg, S. , T. Garland , and A. Ives . 2003. “Testing for Phylogenetic Signal in Comparative Data: Behavioral Traits Are More Labile.” Evolution 57: 717–745. 12778543 10.1111/j.0014-3820.2003.tb00285.x

[ece371616-bib-0006] Carvalho, E. C. , B. C. Souza , M. S. Silva , et al. 2023. “Xylem Anatomical Traits Determine the Variation in Wood Density and Water Storage of Plants in Tropical Semiarid Climate.” Flora 298: 152–185.

[ece371616-bib-0007] Castillo‐Figueroa, D. , A. González‐Melo , and J. Posada . 2023. “Wood Density Is Related to Aboveground Biomass and Productivity Along a Successional Gradient in Upper Andean Tropical Forests.” Frontiers in Plant Science 14, no. 1: 276–424. 10.3389/fpls.2023.1276424 PMC10665531 38023915

[ece371616-bib-0008] Chu, H. , and C. Farrell . 2022. “Fast Plants Have Water‐Use and Drought Strategies That Balance Rainfall Retention and Drought Survival on Green Roofs.” Ecological Applications 32, no. 1: e02486. 34674341 10.1002/eap.2486

[ece371616-bib-0009] Clements, R. , N. S. Sodhi , M. Schilthuizen , and P. K. Ng . 2006. “Limestone Karsts of Southeast Asia: Imperiled Arks of Biodiversity.” Bioscience 56, no. 9: 733–742.

[ece371616-bib-0010] Cornwell, W. , L. Schwilk , and D. Ackerly . 2006. “A Trait‐Based Test for Habitat Filtering: Convex Hull Volume.” Ecology 87, no. 6: 1465–1471. 16869422 10.1890/0012-9658(2006)87[1465:attfhf]2.0.co;2

[ece371616-bib-0011] Dani, R. , P. Divakar , and C. Baniya . 2023. “Diversity and Composition of Plants Species Along Elevational Gradient: Research Trends.” Biodiversity and Conservation 32, no. 8: 2961–2980.

[ece371616-bib-0012] Day, M. , and P. Urich . 2000. “An Assessment of Protected Karst Landscapes in Southeast Asia.” Cave and Karst Science 27, no. 2: 61–70.

[ece371616-bib-0013] De Guzman, M. E. , A. Acosta‐Rangel , K. Winter , F. C. Meinzer , D. Bonal , and L. S. Santiago . 2020. “Hydraulic Traits of Neotropical Canopy Liana and Tree Species Across a Broad Range of Wood Density: Implications for Predicting Drought Mortality With Models.” Tree Physiology 41, no. 1: 24–34. 10.1093/treephys/tpaa106 32803244

[ece371616-bib-0081] de Souza, A. L. T. , D. G. Fonseca , R. A. Libório , et al. 2013. “Influence of Riparian Vegetation and Forest Structure on the Water Quality of Rural Low‐Order Streams in SE Brazil.” Forest Ecology and Management 298: 12–18.

[ece371616-bib-0014] Dirzo, R. , and K. Boege . 2008. “Patterns of Herbivory and Defense in Tropical Dry and Rain Forests.” In Tropical Forest Community Ecology, 63–78. Wiley.

[ece371616-bib-0015] Dong, N. , I. Prentice , I. Wright , et al. 2020. “Components of Leaf‐Trait Variation Along Environmental Gradients.” New Phytologist 228, no. 1: 82–94. 32198931 10.1111/nph.16558

[ece371616-bib-0016] E‐Vojtkó, A. , F. de Bello , Z. Lososová , and L. Götzenberger . 2023. “Phylogenetic Diversity Is a Weak Proxy for Functional Diversity but They Are Complementary in Explaining Community Assembly Patterns in Temperate Vegetation.” Journal of Ecology 111, no. 10: 2218–2230.

[ece371616-bib-0017] Firn, J. , J. M. McGree , E. Harvey , et al. 2019. “Leaf Nutrients, Not Specific Leaf Area, Are Consistent Indicators of Elevated Nutrient Inputs.” Nature Ecology & Evolution 3, no. 3: 400–406. 30718853 10.1038/s41559-018-0790-1

[ece371616-bib-0018] Frazer, G. W. 1999. Gap Light Analyzer (GLA), Version 2.0: Imaging Software to Extract Canopy Structure and Gap Light Transmission Indices From True‐Colour Fisheye Photographs, Users Manual and Program Documentation. Simon Fraser University, Institute of Ecosystem Studies.

[ece371616-bib-0019] Funk, J. , S. Kimball , M. Nguyen , et al. 2023. “Interacting Ecological Filters Influence Success and Functional Composition in Restored Plant Communities Over Time.” Ecological Applications 33, no. 6: e2899. 37335271 10.1002/eap.2899

[ece371616-bib-0020] Garcia, M. , J. Hu , T. Domingues , et al. 2021. “Local Hydrological Gradients Structure High Intraspecific Variability in Plant Hydraulic Traits in Two Dominant Central Amazonian Tree Species.” Journal of Experimental Botany 73, no. 3: 939–952. 10.1093/jxb/erab432 34545938

[ece371616-bib-0021] Garnier, E. , G. Laurent , A. Bellmann , et al. 2001. “Consistency of Species Ranking Based on Functional Leaf Traits.” New Phytologist 152, no. 1: 69–83. 35974476 10.1046/j.0028-646x.2001.00239.x

[ece371616-bib-0022] Garnier, E. , S. Lavorel , P. Ansquer , et al. 2007. “Assessing the Effects of Land‐Use Change on Plant Traits, Communities and Ecosystem Functioning in Grasslands: A Standardized Methodology and Lessons From an Application to 11 European Sites.” Annals of Botany 99, no. 5: 967–985. 17085470 10.1093/aob/mcl215 PMC2802906

[ece371616-bib-0023] Grenié, M. , and H. Gruson . 2023. “Fundiversity: A Modular R Package to Compute Functional Diversity Indices.” Ecography 2023, no. 3: e06585.

[ece371616-bib-0024] Guo, Y. , J. Lu , S. Franklin , et al. 2013. “Spatial Distribution of Tree Species in a Species‐Rich Subtropical Mountain Forest in Central China.” Canadian Journal of Forest Research 43, no. 9: 826–835.

[ece371616-bib-0025] Han, T. , H. Ren , D. Hui , et al. 2023. “Dominant Ecological Processes and Plant Functional Strategies Change During the Succession of a Subtropical Forest.” Ecological Indicators 146: 109–885.

[ece371616-bib-0026] He, G. , X. Zhao , and M. Yu . 2021. “Exploring the Multiple Disturbances of Karst Landscape in Guilin World Heritage Site, China.” Catena 203: 105–349.

[ece371616-bib-0027] He, J. , Y. J. Yan , X. S. Yi , Y. Wang , and Q. H. Dai . 2021. “Soil Heterogeneity and Its Interaction With Plants in Karst Areas.” Ying Yong Sheng Tai Xue Bao, Journal of Applied Ecology 32, no. 6: 2249–2258. 34212631 10.13287/j.1001-9332.202106.006

[ece371616-bib-0028] Holden, E. , and J. Cahill . 2024. “Plant Trait Dissimilarity Increases Competitive Interactions Among Co‐Occurring Plants.” Functional Ecology 38, no. 7: 1464–1474.

[ece371616-bib-0029] Huang, Y. , X. Zhang , R. Zang , et al. 2018. “Functional Recovery of a Subtropical Evergreen‐Deciduous Broadleaved Mixed Forest Following Clear Cutting in Central China.” Scientific Reports 8, no. 1: 16–458. 30405174 10.1038/s41598-018-34896-5 PMC6220334

[ece371616-bib-0030] Hubbell, S. P. 2005. “Neutral Theory in Community Ecology and the Hypothesis of Functional Equivalence.” Functional Ecology 19, no. 1: 166–172.

[ece371616-bib-0031] Jian, M. , X. Gao , W. Wang , C. Cen , and J. Yang . 2025. “Changes in the Association Between Soil Characteristics and Woody Plant Diversity Following the Transformation of Karst Mountainous Forests Into Urban Parks.” Soil Ecology Letters 7, no. 2: 240–285.

[ece371616-bib-0032] Jiang, Y. , Z. Chen , H. Lin , et al. 2024. “Trait‐Based Community Assembly and Functional Strategies Across Three Subtropical Karst Forests, Southwestern China.” Frontiers in Plant Science 15, no. 1: 451–981. 10.3389/fpls.2024.1451981 PMC11417004 39315372

[ece371616-bib-0033] Jiang, Y. , R. Zang , X. Lu , et al. 2015. “Effects of Soil and Microclimatic Conditions on the Community‐Level Plant Functional Traits Across Different Tropical Forest Types.” Plant and Soil 390, no. 1: 351–367.

[ece371616-bib-0034] Jin, Y. , and H. Qian . 2019. “V.PhyloMaker: An R Package That Can Generate Very Large Phylogenies for Vascular Plants.” Ecography 42, no. 8: 1353–1359.

[ece371616-bib-0035] Kembel, S. , P. Cowan , M. Helmus , et al. 2010. “Picante: R Tools for Integrating Phylogenies and Ecology.” Bioinformatics 26, no. 11: 1463–1464. 20395285 10.1093/bioinformatics/btq166

[ece371616-bib-0037] Kraft, N. J. , W. K. Cornwell , C. O. Webb , and D. D. Ackerly . 2007. “Trait Evolution, Community Assembly, and the Phylogenetic Structure of Ecological Communities.” American Naturalist 170, no. 2: 271–283. 10.1086/519400 17874377

[ece371616-bib-0038] Kremer, C. , and C. Klausmeier . 2013. “Coexistence in a Variable Environment: Eco‐Evolutionary Perspectives.” Journal of Theoretical Biology 339: 14–25. 23702333 10.1016/j.jtbi.2013.05.005

[ece371616-bib-0039] LaFrankie, J. 1995. Initial Findings From Lambir: Trees, Soils and Community Dynamics, 5. Center for Tropical Forest Science.

[ece371616-bib-0040] Lavorel, S. , and E. Garnier . 2002. “Predicting Changes in Community Composition and Ecosystem Functioning From Plant Traits: Revisiting the Holy Grail.” Functional Ecology 16, no. 5: 545–556.

[ece371616-bib-0041] Li, X. , X. Zhao , Y. Tsujii , et al. 2022. “Links Between Leaf Anatomy and Leaf Mass Per Area of Herbaceous Species Across Slope Aspects in an Eastern Tibetan Subalpine Meadow.” Ecology and Evolution 12, no. 6: e8973. 35784019 10.1002/ece3.8973 PMC9163673

[ece371616-bib-0042] Li, Y. , W. Bao , F. Bongers , et al. 2019. “Drivers of Tree Carbon Storage in Subtropical Forests.” Science of the Total Environment 654: 684–693. 30448659 10.1016/j.scitotenv.2018.11.024

[ece371616-bib-0043] Lima, A. L. , M. J. Rodal , C. C. Castro , et al. 2021. “Phenology of High‐ and Low‐Density Wood Deciduous Species Responds Differently to Water Supply in Tropical Semiarid Regions.” Journal of Arid Environments 193: 104–594.

[ece371616-bib-0045] Liu, M. , L. Xu , R. Mu , G. Zhang , R. Yu , and L. Li . 2023. “Plant Community Assembly of Alpine Meadow at Different Altitudes in Northeast Qinghai‐Tibet Plateau.” Ecosphere 14, no. 1: e4354.

[ece371616-bib-0046] Liu, W. , L. Zheng , and D. Qi . 2020. “Variation in Leaf Traits at Different Altitudes Reflects the Adaptive Strategy of Plants to Environmental Changes.” Ecology and Evolution 10, no. 15: 8166–8175. 32788969 10.1002/ece3.6519 PMC7417217

[ece371616-bib-0047] Ma, J. , X. Wang , and F. Hou . 2024. “A General Pattern of Plant Traits and Their Relationships With Environmental Factors and Microbial Life‐History Strategies.” Science of the Total Environment 931: 172–670. 10.1016/j.scitotenv.2024.172670 38679109

[ece371616-bib-0048] Mayfield, M. , and J. Levine . 2010. “Opposing Effects of Competitive Exclusion on the Phylogenetic Structure of Communities: Phylogeny and Coexistence.” Ecology Letters 13: 1085–1093. 20576030 10.1111/j.1461-0248.2010.01509.x

[ece371616-bib-0049] Maynard, D. , L. Bialic‐Murphy , C. Zohner , et al. 2022. “Global Relationships in Tree Functional Traits.” Nature Communications 13, no. 1: 3185. 10.1038/s41467-022-30888-2 PMC9177664 35676261

[ece371616-bib-0050] Medeiros, M. , C. L. Wright , A. L. de Lima , et al. 2024. “Divergent Hydraulic Strategies of Two Deciduous Tree Species to Deal With Drought in the Brazilian Semi‐Arid Region.” Trees 38, no. 3: 681–694.

[ece371616-bib-0051] Meng, L. , Y. Li , L. Chen , et al. 2024. “Variations in Species Diversity Patterns and Community Assembly Rules Among Vegetation Types in the Karst Landscape.” Frontiers in Plant Science 15, no. 1: 338–596. 10.3389/fpls.2024.1338596 PMC10917898 38455729

[ece371616-bib-0052] Mertens, D. , K. Bouwmeester , and E. Poelman . 2021. “Intraspecific Variation in Plant‐Associated Herbivore Communities Is Phylogenetically Structured in Brassicaceae.” Ecology Letters 24, no. 11: 2314–2327. 34331409 10.1111/ele.13852 PMC9291228

[ece371616-bib-0053] Nawaz, M. , J. Sun , S. Shabbir , et al. 2023. “A Review of Plants Strategies to Resist Biotic and Abiotic Environmental Stressors.” Science of the Total Environment 900: 165–832. 10.1016/j.scitotenv.2023.165832 37524179

[ece371616-bib-0054] Nosrati, H. , S. Mirtajadini , and M. Jahanshahi . 2023. “Phylogenetic Structure of Plant Community, and Its Relationship With Environmental Components,” Authorea Preprints.

[ece371616-bib-0055] Ogasa, M. , N. H. Miki , Y. Murakami , and K. Yoshikawa . 2013. “Recovery Performance in Xylem Hydraulic Conductivity Is Correlated With Cavitation Resistance for Temperate Deciduous Tree Species.” Tree Physiology 33, no. 4: 335–344. 23492871 10.1093/treephys/tpt010

[ece371616-bib-0056] Pantaleão, L. C. , L. F. de Moraes , F. V. Cesário , et al. 2024. “Linking Plant Functional Traits to Soil Properties in Tropical Forest Restoration.” Forest Ecology and Management 563: 121–976.

[ece371616-bib-0057] Perez‐Harguindeguy, N. , S. Diaz , E. Garnier , et al. 2016. “Corrigendum to: New Handbook for Standardised Measurement of Plant Functional Traits Worldwide.” Australian Journal of Botany 64: 715.

[ece371616-bib-0058] Pyron, R. , G. Costa , M. Patten , et al. 2015. “Phylogenetic Niche Conservatism and the Evolutionary Basis of Ecological Speciation.” Biological Reviews 90, no. 4: 1248–1262. 25428167 10.1111/brv.12154

[ece371616-bib-0059] Reich, P. 2014. “The World‐Wide “Fast–Slow” Plant Economics Spectrum: A Traits Manifesto.” Journal of Ecology 102, no. 2: 275–301.

[ece371616-bib-0060] Rowland, L. , J. Ramírez‐Valiente , I. Hartley , J. A. Ramírez‐Valiente , I. P. Hartley , and M. Mencuccini . 2023. “How Woody Plants Adjust Above‐ and Below‐Ground Traits in Response to Sustained Drought.” New Phytologist 239, no. 4: 1173–1189. 37306017 10.1111/nph.19000

[ece371616-bib-0061] Sánchez de Dios, R. , L. DeSoto , B. Cortón , and L. Hernández . 2023. “The Renaissance of Mixed Forests? New Insights Into Shifts in Tree Dominance and Composition Following Centuries of Human‐Induced Simplification of Iberian Forests.” Ecosystems 26, no. 6: 1159–1172.

[ece371616-bib-0063] Subedi, S. , J. Hogan , M. Ross , et al. 2019. “Evidence for Trait‐Based Community Assembly Patterns in Hardwood Hammock Forests.” Ecosphere 10, no. 12: e02956.

[ece371616-bib-0064] Swenson, N. , and B. Enquist . 2009. “Opposing Assembly Mechanisms in a Neotropical Dry Forest: Implications for Phylogenetic and Functional Community Ecology.” Ecology 90, no. 8: 2161–2170. 19739378 10.1890/08-1025.1

[ece371616-bib-0065] Tilman, D. 1982. Resource Competition and Community Structure (MPB‐17). Princeton University Press. 7162524

[ece371616-bib-0066] Tokeshi, M. 1990. “Niche Apportionment or Random Assortment: Species Abundance Patterns Revisited.” Journal of Animal Ecology 59: 1129–1146.

[ece371616-bib-0067] Wang, Q. , C. Wang , and J. Wan . 2022. “Relationships Between Topographic Variation and Plant Functional Trait Distribution Across Different Biomes.” Flora 293: 152116.

[ece371616-bib-0068] Wang, Z. , J. Arratia , T. Yan , C. Zhang , and A. Chiarucci . 2024. “Integrative Framework of Multiple Processes to Explain Plant Productivity–Richness Relationships.” Frontiers in Ecology and Evolution 12: 1,332,985.

[ece371616-bib-0069] Watson, J. , ed. 1997. Guidelines for Cave and Karst Protection. IUCN.

[ece371616-bib-0070] Webb, C. 2000. “Exploring the Phylogenetic Structure of Ecological Communities: An Example for Rain Forest Trees.” American Naturalist 156: 145–155. 10.1086/303378 10856198

[ece371616-bib-0071] Wright, I. , P. Reich , M. Westoby , et al. 2004. “The Worldwide Leaf Economics Spectrum.” Nature 428, no. 6985: 821–827. 15103368 10.1038/nature02403

[ece371616-bib-0072] Xia, L. , L. Cao , Y. Yang , et al. 2023. “Integrated Biochar Solutions Can Achieve Carbon‐Neutral Staple Crop Production.” Nature Food 4: 236–246. 37118263 10.1038/s43016-023-00694-0

[ece371616-bib-0073] Xu, S. , H. Su , S. Ren , J. Hou , and Y. Zhu . 2023. “Functional Traits and Habitat Heterogeneity Explain Tree Growth in a Warm Temperate Forest.” Oecologia 203, no. 3: 371–381. 37910255 10.1007/s00442-023-05471-1

[ece371616-bib-0074] Xue, J. , Z. Zhou , and Y. Wu . 2022. “Research Progresses on Ecological Remediation of the Degraded Soil in Karst Rocky Desertification Mountainous Areas.” Journal of Nanjing Forestry University 46, no. 6: 135.

[ece371616-bib-0075] Yang, J. , Y. Gao , C. Zhao , and H. Chen . 2023. “Leaf Phenotypic Plasticity and Integration Balance Plant Adaptation to Water Table Decline: A Mesocosm Experiment.” Plant and Soil 497, no. 1–2: 611–627.

[ece371616-bib-0077] Zakharova, L. , K. M. Meyer , and M. Seifan . 2019. “Trait‐Based Modelling in Ecology: A Review of Two Decades of Research.” Ecological Modelling 407: 108–703.

[ece371616-bib-0078] Zeng, X. , X. Xu , R. Yi , F. Zhong , and Y. Zhang . 2021. “Sap Flow and Plant Water Sources for Typical Vegetation in a Subtropical Humid Karst Area of Southwest China.” Hydrological Processes 35, no. 3: e14090.

[ece371616-bib-0079] Zhang, W. C. , W. Wu , J. W. Li , and H. B. Liu . 2023. “Climate and Topography Controls on Soil Water‐Stable Aggregates at Regional Scale: Independent and Interactive Effects.” Catena 228: 107–170.

[ece371616-bib-0080] Zhao, J. , Y. Zhang , J. Xu , et al. 2022. “Strong Environmental Filtering Based on Hydraulic Traits Occurring in the Lower Water Availability of Temperate Forest Communities.” Frontiers in Plant Science 12: 698–878. 10.3389/fpls.2021.698878 PMC8811132 35126402

