# Supplementary material for: Untangling Community Assembly Through Functional Traits and Phylogenetic Alpha Diversity in Subtropical Karst Forests
Source: Ecol Evol. 2025 Jun 26;15(7):e71616. doi: 10.1002/ece3.71616 (PMC12202465; doi:10.1002/ece3.71616)
Supplement: Supplementary file 1 — Appendix S1. [file ECE3-15-e71616-s004.docx]

**Untangling Community Assembly through Functional Traits and Phylogenetic Alpha Diversity in Subtropical Karst Forests**

Shichu Liang, Miao Dong, Yong Jiang, Daniel F. Petticord, Junwei Li, Jianghui Long, Quan Su

**Appendix Table S1**

Species importance value was used to quantify the dominance of species in different forest systems. It was calculated as the average of relative density, relative basal area, and relative frequency (Liu et al., 2014). The formula is as follows:

$$IV=\frac{(RD+RA+RF)}{3}\times100\%$$

where *IV* is the importance value, *RD* is the relative dominance, *RA* is the relative density, *RF* is the relative frequency.

**Table S1.** Difference on top 20 dominant species importance at each forest type.

|  | Deciduous forests | | Mixed forests | | Evergreen forests | |
| --- | --- | --- | --- | --- | --- | --- |
|  | Species | IV (%) | Species | IV (%) | Species | IV (%) |
| 1 | *Celtis sinensis* | 28.2 | *Quercus glauca* | 19.9 | Quercus glauca | 35.5 |
| 2 | *Mallotus repandus* | 10.1 | *Zelkova schneideriana* | 9.7 | *Mallotus philippensis* | 9.8 |
| 3 | *Choerospondias axillaris* | 9.1 | *Boniodendron minus* | 8.6 | *Pittosporum planilobum* | 6.1 |
| 4 | *Chimonanthus nitens* | 7.1 | *Mallotus philippensis* | 4.8 | *Decaspermum parviflorum* | 4.2 |
| 5 | *Cornus wilsoniana* | 4.8 | *Cinnamomum saxatile* | 4.1 | *Albizia julibrissin* | 3.8 |
| 6 | *Boniodendron minus* | 4.8 | *Pittosporum planilobum* | 3.9 | *Murraya exotica* | 2.7 |
| 7 | *Ficus microcarpa* | 4.3 | *Callicarpa bodinieri* | 3.4 | *Diplospora dubia* | 1.9 |
| 8 | *Clausena anisum-olens* | 3.9 | *Garcinia paucinervis* | 3.2 | *Flueggea suffruticosa* | 1.9 |
| 9 | *Triadica rotundifolia* | 2.8 | *Triadica rotundifolia* | 3.1 | *Ficus erecta* | 1.9 |
| 10 | *Mallotus philippensis* | 2.7 | *Radermachera sinica* | 2.7 | *Sinoadina racemosa* | 1.7 |
| 11 | *Sophora prazeri* | 2.3 | *Sinoadina racemosa* | 2.6 | *Cinnamomum saxatile* | 1.7 |
| 12 | *Murraya exotica* | 2.3 | *Celtis biondii* | 2.5 | *Platyosprion platycarpum* | 1.6 |
| 13 | *Sinoadina racemosa* | 1.8 | *Rhamnus lamprophylla* | 2.3 | *Loropetalum chinense* | 1.5 |
| 14 | *Vitex negundo* | 1.5 | *Bridelia retusa* | 2.2 | *Triadica rotundifolia* | 1.5 |
| 15 | *Celtis biondii* | 1.4 | *Lindera megaphylla* | 2.9 | *Celtis biondii* | 1.2 |
| 16 | *Pittosporum planilobum* | 1.4 | *Machilus calcicola* | 2.0 | *Decaspermum gracilentum* | 1.2 |
| 17 | *Radermachera sinica* | 1.2 | *Pistacia chinensis* | 1.8 | *Lithocarpus litseifolius* | 1.1 |
| 18 | *Photinia serratifolia* | 1.2 | *Pteroceltis tatarinowii* | 1.5 | *Millettia pulchra* | 1.0 |
| 19 | *Psydrax dicocca* | 1.1 | *Ficus tinctoria* | 1.3 | *Alchornea trewioides* | 0.9 |
| 20 | *Ulmus pumila* | 1.0 | *Croton euryphyllus* | 1.2 | *Maclura cochinchinensis* | 0.8 |
| Total | Evergreen dominant species | 30.1 | Evergreen dominant species | 40.2 | Evergreen dominant species | 60.8 |
|  | Deciduous dominant species | 63.0 | Deciduous dominant species | 42.7 | Deciduous dominant species | 22.1 |

**Appendix Figure S1**

To determine whether significant differences exist between forest types, we conducted an Analysis of Similarities (ANOSIM; Clarke, 1993) using Bray-Curtis distances. ANOSIM is a robust, non-parametric method for testing dissimilarities among groups of samples (Somerfield et al., 2021). The test generates an R-statistic, which quantifies the mean rank difference between groups. A higher R-value indicates stronger differentiation between communities. These analyses were performed using the ‘vegan’ package (version 2.0.6; Oksanen et al., 2013) in R software (version 4.0.2; R Core Team, 2013).Figure S1 clearly revealed significant difference in species composition between across all forest types (R= 0.968, *p* = 0.001) (Figure S1A) and in pair-wise comparisons of forest types (*p* = 0.001, R=0.982, R=0.998 and R=0.994) between groups (Figure S1B, C, D).


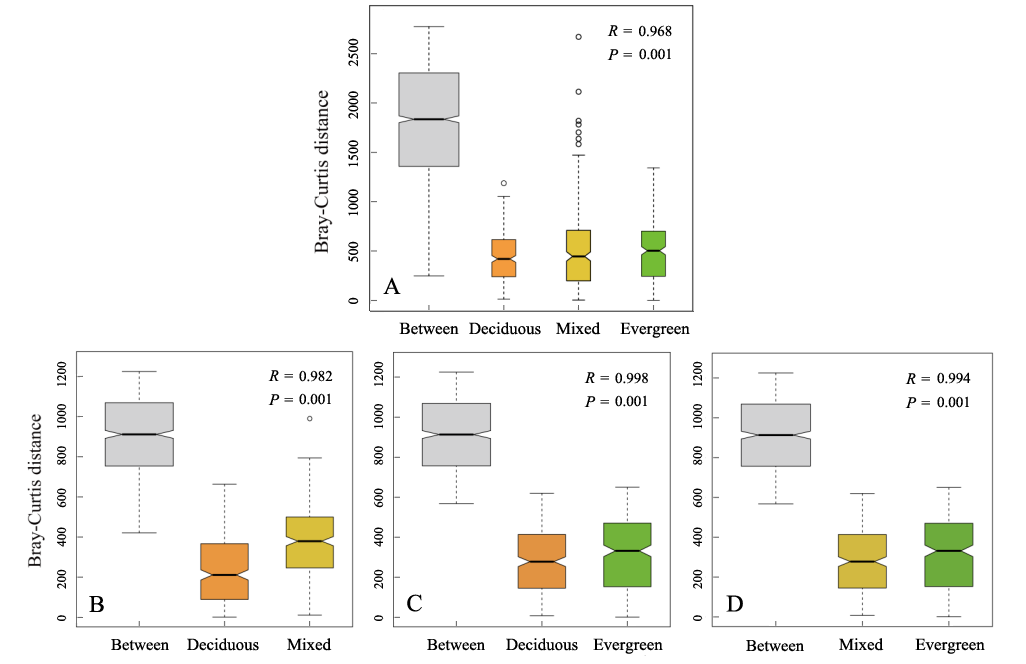


**Figure S1.** Analysis of similarities between across all forest types (A)and in pair-wise comparisons of forest types(B,C,D).

**Appendix Figure S2**

Further, we performed Correspondence Analysis (CA) method using the species importance values data to distinguish this three forest types. Figure S2 showed the sample plots and species data can be divided into three distinct forest types.


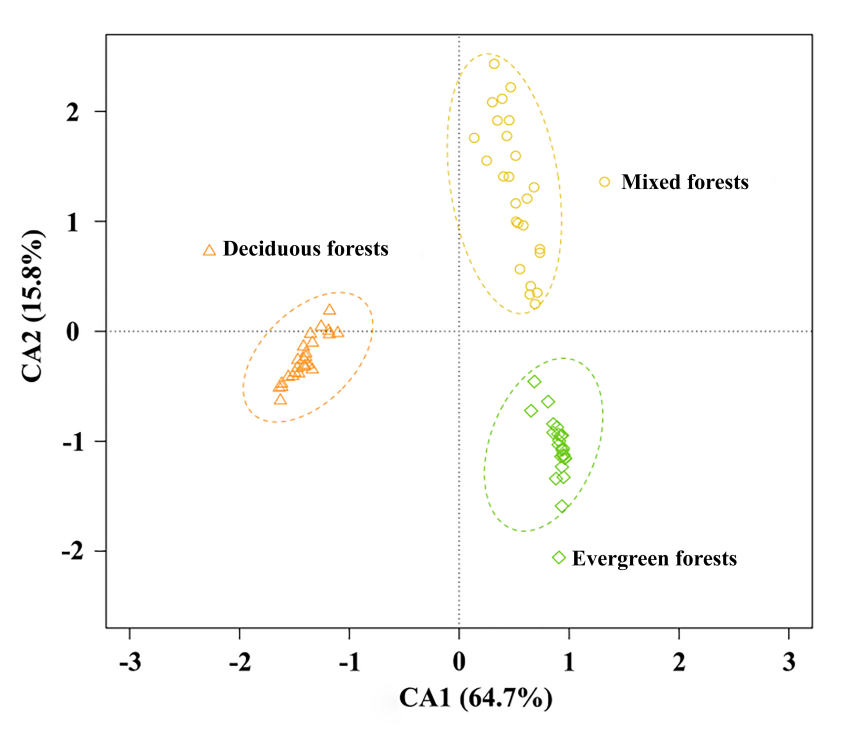


**Figure S2.** Two-dimensional CA ordination diagram of plots for three forest types. Orange triangles represent deciduous forests; yellow circles represent mixed forests; green diamonds evergreen forests. The first CA ordination axis accounted for 64.7 % of the total variation, and together the first two CA ordination axes accounted for 80.5% of the total variation.

**Appendix Table S2**

Species were categorized into three DBH size classes based on Li et al. (2009). Small trees: 1cm≤DBH≤4 cm; Medium trees: 4cm<DBH≤8 cm; Large trees:DBH>8 cm (Table S2 provides further details on species distribution across size classes).

**Table S2.** The number of diameter classes among tree species in three forest types

| **Diameter division of tree species** | **Deciduous forests** | | **Mixed forests** | | **Evergreen forests** | |
| --- | --- | --- | --- | --- | --- | --- |
|  | **Species** | **Number** | **Species** | **Number** | **Species** | **Number** |
| Small trees (1 cm ≤ DBH ≤ 4 cm) | 43 | 2047 | 32 | 713 | 43 | 3489 |
| Medium trees (4 cm < DBH ≤ 8 cm) | 11 | 530 | 26 | 375 | 20 | 1786 |
| Large trees (DBH > 8cm) | 9 | 363 | 33 | 520 | 21 | 1808 |

**Appendix Figure S3**


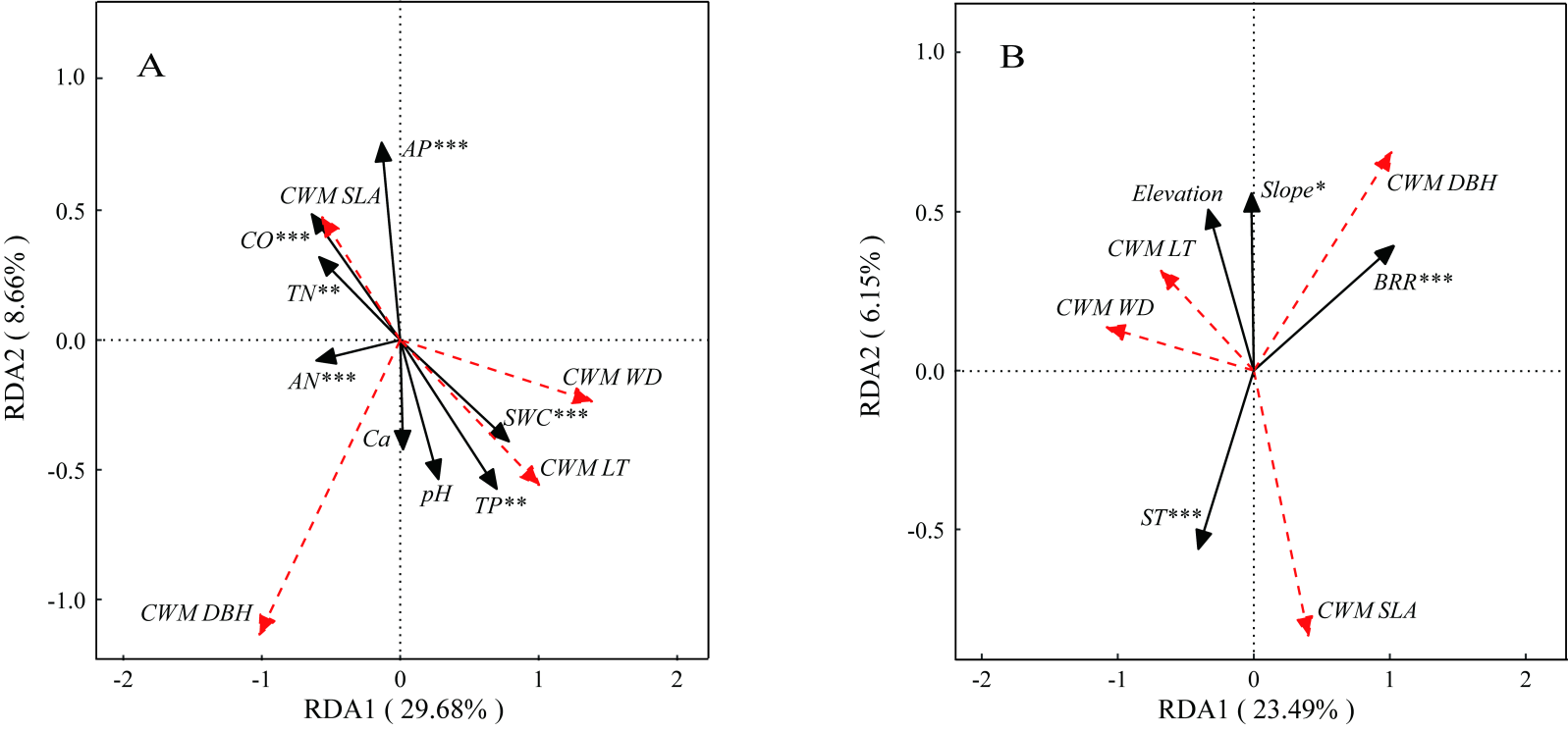
We used redundancy analysis (RDA) to examine the relationship between functional traits and environmental factors across all forest types. The results indicate that available phosphorus (AP), canopy openness (CO), soil water content (SWC), total nitrogen (TN), available nitrogen (AN), and total phosphorus (TP) are significantly correlated with four community-weighted mean (CWM) functional traits (SLA, LT, WD, and DBH) (Figure S3A). Additionally, slope, soil thickness, and bare rock ratio (BRR) were significantly associated with these traits (Figure S3B). These findings underscore the strong influence of environmental variables on community-level functional trait variation.

**Figure S3.** The relationship between CWM traits and environmental factors by redundancy analysis (RDA) across all forest types. The dashed line with red arrows represent the four CWM functional traits and the solid line with black arrows represent environmental factors. *indicate level of significance (**p*˂0.05; ***p*˂0.01; ****p*˂0.001).

**Appendix Figure S4**

We assessed differences in three key functional traits (SLA, LT, and DBH) among three dominant plant families—Euphorbiaceae, Fagaceae, and Leguminosae—using one-way ANOVA with post-hoc multiple comparisons in evergreen forests,SLA was significantly higher in Leguminosae compared to Euphorbiaceae and Fagaceae (*p* < 0.05; Figure S4A). LT was significantly higher in Fagaceae than in Euphorbiaceae and Leguminosae (*p* < 0.05; Figure S4B). DBH was significantly higher in Fagaceae compared to Euphorbiaceae and Leguminosae (*p* < 0.05; Figure S4C). These results suggest divergent ecological strategies among plant families, with Leguminosae favoring resource acquisition (high SLA), while Fagaceae prioritize structural investment (high LT and DBH).


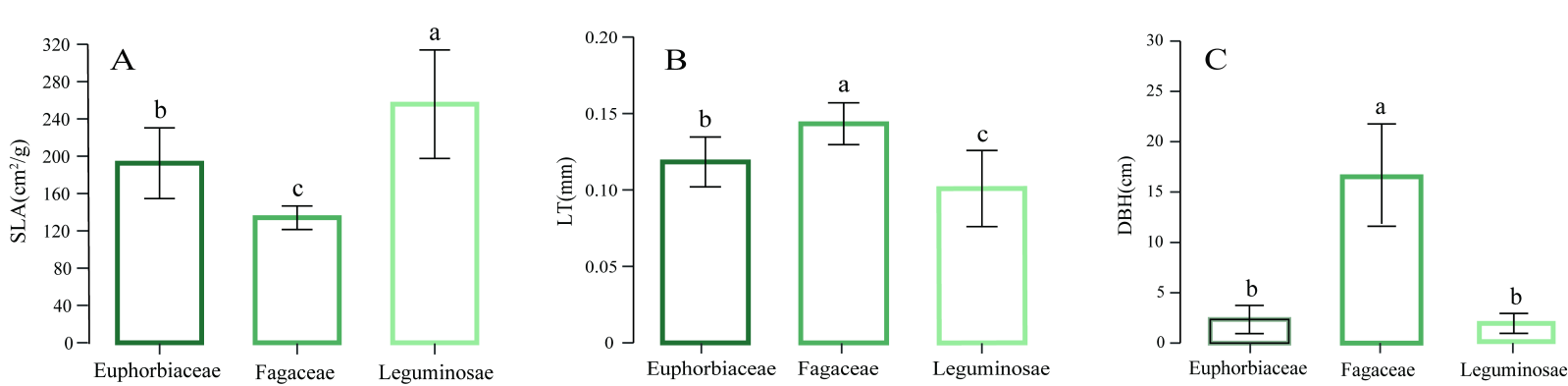


**Figure S4.** The difference among three functional traits in three plant families in evergreen forests. Different lowercase letters indicate significant difference (*p* < 0.05).

**Appendix Figure S5**

We applied Canonical Correspondence analysis (CCA) based on environmental data matrix and traits matrix for sampling scale of 20m×20m. We found that TP, TN, CO and Slope significantly influenced four CWMtraits in deciduous forests(Figure S5A) while Convexity, CO and BRR had a strong impact on them in mixed forests(Figure S5B). In evergreen forests, SWC, ST and TN had a strong impact on four CWMtraits(Figure S5C) .


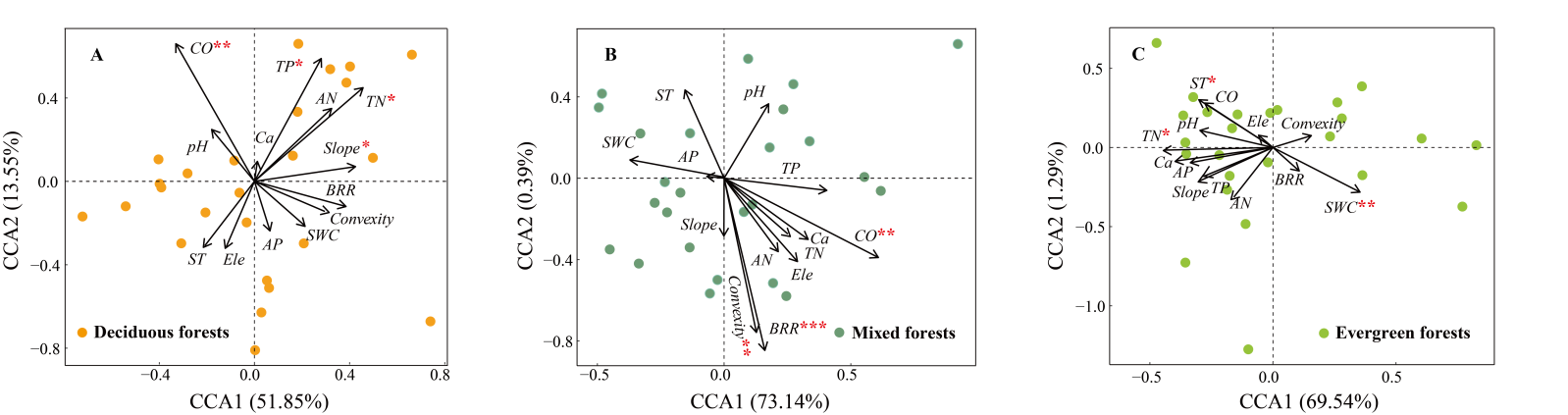


**Figure S5.** The relationship between environmental variables and CWM_traits_ in three forest types by Canonical Correspondence analysis CCA ordination (**p*˂0.05; ***p*˂0.01; ****p*˂0.001).

**References**

Clarke K R (1993). Non-parametric multivariate analyses of changes in community structure. Australian Journal of Ecology, 18, 117–143.

Li L, Huang Z L, Ye W H, et al. (2009). Spatial distributions of tree species in a subtropical forest of China. Oikos, 118: 495-502.

Liu C, Xiang W H, Lei P F, et al. 2014. Standing fine root mass and production in four Chinese subtropical forests along a succession and species diversity gradient. Plant Soil , 376, 445–459.

Somerfield P J, Clarke K R, Gorley R N (2021). A generalised analysis of similarities (ANOSIM) statistic for designs with ordered factors. Austral Ecology , 46, 901–910.

Oksanen J, Blanchet F G, Kindt R, et al. (2013). Community ecology package. R package version , 2, 321–326.

Pinheiro J, Bates D, DebRoy S, et al. 2009. R Development Core Team, 2013. nlme. Linear and nonlinear miexed effects models. 3, 1-110.
